# Mathematical model of blunt injury to the vascular wall via formation of rouleaux and changes in local hemodynamic and rheological factors. Implications for the mechanism of traumatic myocardial infarction

**DOI:** 10.1186/1742-4682-2-13

**Published:** 2005-03-30

**Authors:** Rovshan M Ismailov

**Affiliations:** 1 Department of Epidemiology, Graduate School of Public Health, University of Pittsburgh, Pittsburgh, PA 15213, USA

## Abstract

**Background:**

Blood viscosity is fundamentally important in clinical practice yet the apparent viscosity at very low shear rates is not well understood. Various conditions such as blunt trauma may lead to the appearance of zones inside the vessel where shear stress equals zero. The aim of this research was to determine the blood viscosity and quantitative aspects of rouleau formation from erythrocytes at yield velocity (and therefore shear stress) equal to zero. Various fundamental differential equations and aspects of multiphase medium theory have been used. The equations were solved by a method of approximation. Experiments were conducted in an aerodynamic tube.

**Results:**

The following were determined: (1) The dependence of the viscosity of a mixture on volume fraction during sedimentation of a group of particles (forming no aggregates), confirmed by published experimental data on the volume fractions of the second phase (*f*_2_) up to 0.6; (2) The dependence of the viscosity of the mixture on the volume fraction of erythrocytes during sedimentation of rouleaux when yield velocity is zero; (3) The increase in the viscosity of a mixture with an increasing erythrocyte concentration when yield velocity is zero; (4) The dependence of the quantity of rouleaux on shear stress (the higher the shear stress, the fewer the rouleaux) and on erythrocyte concentration (the more erythrocytes, the more rouleaux are formed).

**Conclusions:**

This work represents one of few attempts to estimate extreme values of viscosity at low shear rate. It may further our understanding of the mechanism of blunt trauma to the vessel wall and therefore of conditions such as traumatic acute myocardial infarction. Such estimates are also clinically significant, since abnormal values of blood viscosity have been observed in many pathological conditions such as traumatic crush syndrome, cancer, acute myocardial infarction and peripheral vascular disease.

## Introduction

Blood is a liquid-liquid suspension because erythrocytes exhibit fluid-like behavior under certain shear conditions [1]. The dependence of viscosity on shear rate is one of the most widely used rheological measurements [2]. Normal blood also thins when it is sheared, therefore its apparent viscosity is highly sensitive to shear rates below 100 s^-1 ^[2,3].

The objective of this research was to determine blood viscosity at yield velocity (and therefore shear stress) equal to zero. Our previous studies have shown that conditions such as blunt trauma to large vessels may lead to boundary layer separation where du/dy = 0, i.e. to the appearance of zones where shear stress equals zero [4]. A further aim of this research was to evaluate quantitative aspects of rouleau formation from erythrocytes when the yield velocity is equal to zero.

## Methods

Various calculations have been made for the viscosity of a mixture and the coefficient of constraint [5-7]. There is considerable variation in such calculations, resulting from different combinations of phases. This variation apparently reflects the non-Newtonian nature of concentrated viscous disperse mixtures and the insufficiency of the variables *ρ *and *μ *alone (where *ρ *is density and *μ *is viscosity) to determine the mechanical properties of such mixtures. In this regard, experiments over the range of operating parameters are needed for any mixture to determine pressure loss using different rheological models; in particular, the model of a viscous fluid with an effective viscosity coefficient. It must be noted that when *f_2 _*> 0.1 (where *f*_*2 *_is the volume fraction of the second phase), not only the shape and size of the erythrocytes but also the irregular arrangement of the particles and their collisions with each other and with the solid walls have substantial effects on the effective viscosity and other rheological characteristics of the mixture [8,9].

The problems mentioned above have led to studies of group sedimentation at *f*_*2 *_> 0.1 in the interpenetrating model of two- or multi-phase media [10]. These studies usually deal with either high- or low-concentration mixtures. Mechanisms of sedimentation in moderately concentrated mixtures, which are rather common, have not been fully investigated. Mathematical modeling of group sedimentation of particles (in our case, rouleaux) in two-phase interpenetrating media [11] should take into account not only the Stokes force [12] but also other forces that are given in [13]:



where *F*_*12*_^*(A) *^is a buoyancy force, *p- *pressure difference, *χ*^(*m*)^- coefficient of constraint, *ρ*- density of the first phase, *K*^*(μ) *^– coefficient of phase interaction, *μ*_*1 *_and *μ*_*2 *_– viscosities of the first and second phases, *f*_*2 *_– the volume fraction of the second phase. It is also important to calculate *μ*, the viscosity of the blood mixture, which depends on the volume fraction of particles. In this case it is possible to determine the force *F*_*12*_^*(μ)*^. *F*_*12*_^*(μ) *^is a frictional force or Stokes force that results from viscous forces involved in the interaction between phases. *F*_*12*_^*(μ) *^is calculated using the difference between velocities (slippage) *u_1 _- u_2_*, the particle size *a*, the quantities and shapes of inclusions, and the physical properties of the phases (see equation 1). (The effects of the shape and multiplicity of particles, and of some other variables included in the expression for *F*_*12*_^*(μ)*^, are accounted for in coefficients *K*^*(μ)  *^in (1)).

Using all of the above, I shall determine blood viscosity as a variable dependent on a volume fraction of particles. This will allow me to determine blood viscosity at a yield velocity of zero, and the number of rouleaux as a variable dependent on erythrocyte concentration, shear stress and yield velocity.

### Determination of viscosity of a mixture as a variable dependent on volume fraction of particles

Sedimentation of a single particle is based on the Stokes law, according to which a frictional force resulting from the motion of spherical particles with diameter *d *and velocity *V *in a medium of viscosity *μ *is expressed by the equation:



where *a *– radius of particles (inclusions) and *V *– velocity of particle precipitation.

In the general case of a multiphase medium, the frictional force or Stokes force *F*_*12*_^*(μ)*^, which results from viscous forces involved in the interactions between phases, is calculated using the difference between velocities (slippage) *u_1 _- u_2_*, the particle size *a*, the quantity and shape of inclusions, and the physical properties of the phases. Multiphase models are based on the idea of interpenetrating media, where the system of particles is replaced by a mathematical continuum and particle size is considerably less than the distance over which flow conditions may change [11].

The force of gravity acting on a particle is calculated using the specific gravity of the particle; that is:



where *ρ*_1_;*ρ*_2_;*g *are respectively the density of the fluid, the density of the particle, and the acceleration due to gravity.

 is a buoyancy force (Archimedes force);

 is a frictional force or Stokes force.

Force  causes a particle to accelerate. In addition to gravity, the particle is affected by the frictional force, which acts in the opposite direction and has a value directly proportional to the velocity according to the Stokes law. This means that force  and gravity  tend to cancel each other out. Therefore, the motion proceeds with a constant velocity *V *that can be determined from equations (2) and (3):



where *Vs *– velocity of precipitation of a single particle.

Sometimes investigators have to deal with the sedimentation of multiple particles in concentrated mixtures. Formulae for the velocity of sedimentation of particles, dependent on the concentration and velocity of a single particle in an infinite fluid, can be derived using statements from the interpenetrating model [13] and the Euler equation [14]. Assuming that a specific volume has two phases differing in specific gravity, the particles with the greater specific gravity will start moving down a channel, so that a process of mutual penetration occurs.

The flow of the fluid can be expressed by criterion equations:



where *E*_*u *_– Euler number, *A *– coefficient of proportionality, *R*_*e *_– Reynolds number; or:



In the process of sedimentation when the concentration of inclusions is rather high and the particle size is small, flow is laminar; m = - 1 and n = 1 (where m and n are criterion coefficients).

Taking into account data from [13]:



where *S*_*i *_– particle surface area; *f*_1 _– volume fraction of the first phase; *f*_2 _– volume fraction of the second phase

Dividing the continuity equation:

*V*_1_S = *V*_1*i*_*S*_1_

by *S*, I obtain:

*V*_1 _= *f*_1_*V*_1*i*_

where S is the area of the canal section.

Therefore:



Using equations (5) and (2), I can transform the last equation into the Kozeny-Carman formula for restrained sedimentation in a laminar flow:



where *A *lies within the range 80–110.

Dividing equation (7) by the number of particles per unit of volume allows the resistance force applied by the fluid to a single particle to be derived as:



Where F* – resistance force created by the fluid and acting on a single particle, and *χ *– coefficient of resistance for precipitation of multiple particles.

The resistance force applied to a single particle during precipitation in a fluid is known to be [12,15]:



For particles suspended in a fluid:

F* = *F*_12_

therefore from (8) and (9) it follows that:



where *β *– the ratio of the velocity of sedimentation of the group of particles to the velocity of sedimentation of a single particle, and *χ*_*c *_– the coefficient of resistance when precipitating a single particle in an infinite fluid.

From (10), when *f*_1 _→ 1 it follows that:



when the Reynolds numbers are small:



where c – constant.

Therefore, it can be assumed that:



From equations (10) and (11) it follows that:



where:



where *ν *– the coefficient of viscosity.

When the motion is laminar, according to the Stokes law:



Substituting this expression in equation (12), it follows that:



If one considers the sedimentation of a particle in a suspension with viscosity *μ*_*m *_and density *ρ*_*m*_, then the equilibrium equation [13] can be expressed as:





*ρ*_*m *_= *f*_1_*ρ*_1*i *_+ *f*_2_*ρ*_2*i*_

**Figure 1:**
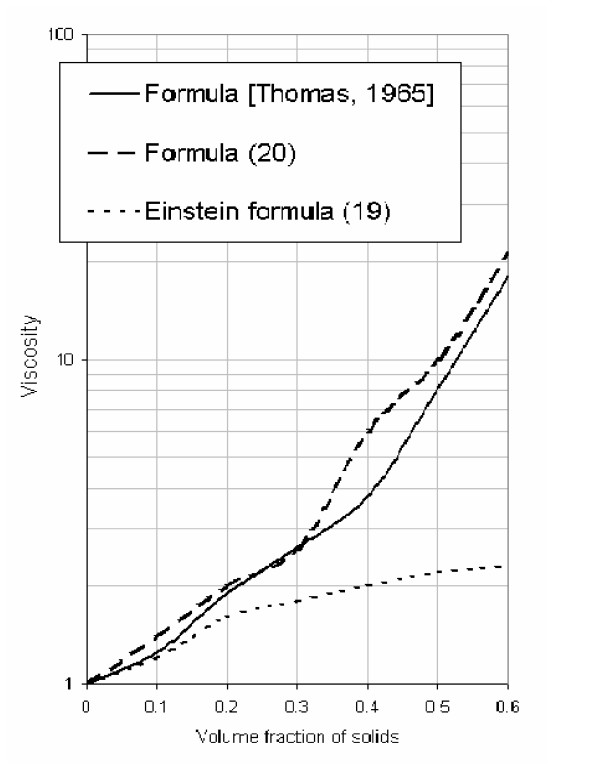
The dependence of a change in relative viscosity on the volume fraction of particles.

**Figure 2:**
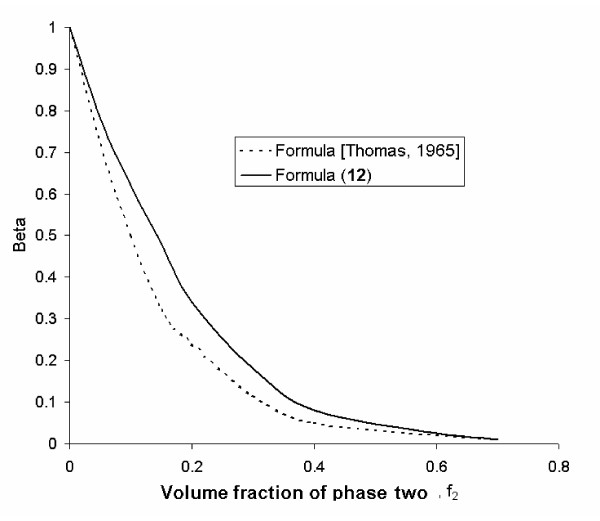
Dependence of relative sedimentation velocity on particle concentration (where *β *is a change in the relative velocity).

Using equations (14), (15) and (3) and the condition *V*_1 _= 0 it follows that:



Substituting the relative velocity equation (13) into equation (17), it follows that:



When *f*_1 _→ 1 and c = 2.5, this reduces to the Einstein formula:



From the calculation given in Figure 1, it follows that equation (18) is consistent with the experimental data (up to *f*_2 _= 0.5 when c = 2.5) obtained by other investigators [6,7] regarding the velocity changes in suspensions for a wide range of fluids and particle sizes as well as particle compositions. Figure 2 shows the relationship between relative sedimentation velocity and particle concentration. The relationship between relative velocity, viscosity and volume fraction is also consistent with experimental data [6,7].

### Determination of viscosity when yield velocity equals zero

The value of viscosity derived in equation (18) describes the sedimentation of solid particles, that is particles that do not form rouleaux. I shall now determine the viscosity of blood when the yield velocity is zero. It is known [16] that if whole blood (in which coagulation is prevented) is placed in a vertically-positioned capillary tube, erythrocytes will aggregate into rouleaux and then sediment. Therefore the viscosity *μ*_*1 *_must be determined in blood that has minimal numbers of rouleaux, and it is necessary to take into account the effect on rouleau sedimentation of erythrocytes that remain suspended. Such a condition occurs when the yield velocity is high (500 – 1000 s^-1^) and the number of rouleaux is minimal. This condition can be expressed by equations (18) or (19) when *f_1  _*→ *1 *and c = 2.5; that is rouleaux do not sediment in plasma but rather in a mixture of erythrocytes, plasma and a certain number of rouleaux.

Calculations made according to equations (18) or (19) when *f*_1 _→ *1 *and c = 2.5 yield the following results:

*μ*_1 _= 6.8 mNsm^-2 ^when concentration of erythrocytes is 28.7%

*μ*_1 _= 8.8 mNsm^-2 ^when concentration of erythrocytes is 48%

*μ*_1 _= 10 mNsm^-2 ^when concentration of erythrocytes is 58.9%

**Figure 3:**
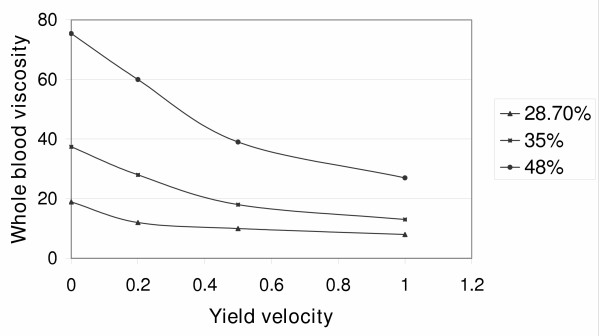
The dependence of viscosity on yield velocity.

These data are consistent with experimental data [16] when the yield velocity ranges from 500 to 1000 s^-1^. Thus, using the effect of the viscosity of the mixture from equations (18) and (19), I can calculate the viscosity of the blood at zero velocity by means of the following equation:



In this equation, when coefficient c = 2.5, there is a minimal number of rouleaux at *μ*_1 _= 3 to 4 mNsm^-2 ^(the value of viscosity when the maximum yield velocity is more than 500 s^-1^). Figure 3, where the viscosity at zero yield velocity is plotted on the Y axis, shows that viscosity increases with increasing concentration. Thus an increase in erythrocyte concentration results in an increase of viscosity.

I shall now determine the shear stress at various concentrations and yield velocities. Table 1 shows that an increase of shear stress causes a decrease of viscosity. Thus, an increase in the concentration of erythrocytes will result in an increase of viscosity and a decrease in shear stress. It can be assumed that a maximal number of rouleaux is formed when the yield velocity is zero, since there are no forces that disassemble them. Then I can determine the number of rouleaux at different values of viscosity and shear stress. Table 2 shows these data and indicates that the main source of rouleaux is the erythrocytes themselves. The higher the erythrocyte concentration, the more rouleaux remain in the blood despite an increase in the forces that destroy them. It is also clear that an increase in shear stress results in a decrease of the number of rouleaux.

**Table 1:** Relationship between shear stress and viscosity

**Yield velocity (s^-1^)**	**The volume fraction of the second phase**	**Viscosity (mNsm^-2^)**	**Shear stress (N/m^2^)**
0.2	28.7	13	0.0026
	35.9	30	0.006
	48	63	0.0126

5	28.7	6	0.03
	35.9	8	0.04
	48	15	0.075

100	28.7	4	0.4
	35.9	5	0.5
	48	6	0.6

500	28.7	3	1.5
	35.9	3	1.5
	48	4	2

**Table 2:** The relationship between erythrocyte concentration and number of rouleaux

**Yield velocity (s^-1^)**	**Concentration %**	**Viscosity (mNsm^-2^)**	**Rouleaux concentration %**	**Concentration of destroyed rouleaux %**	**Shear stress (N/m^2^)**
0.2	28.7	15	65.2	34.8	0.0026
	35.9	30	81	19	0.006
	48	63	83	17	0.0126

5	28.7	6	26	74	0.03
	35.9	8	21.3	78.7	0.04
	48	15	20	80	0.075

**Figure 4:**
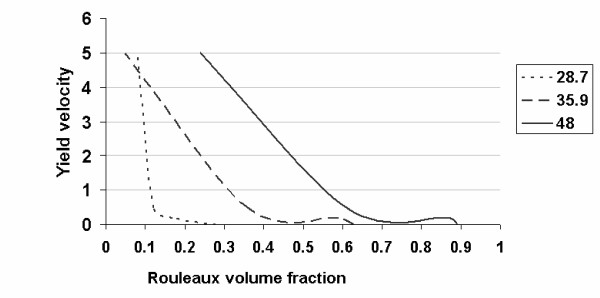
The relationship between the volume fraction of rouleaux and yield velocity.

I can now determine the concentration of rouleaux, assuming that viscosity is determined by the numbers of erythrocytes only at a high yield velocity (since high yield velocities destroy rouleaux). Granted this assumption, the viscosity is determined according to the Einstein equation (18) and (19). Viscosity at decreasing yield velocity is determined by both erythrocytes and newly-formed rouleaux. Then, according to equation (20), I obtain the result presented in Figure 4: the number of rouleaux decreases sharply with increasing yield velocity. Therefore, the number of rouleaux depends on the concentration of erythrocytes.

The quantity of rouleaux depends on shear stress (the higher the shear stress, the lower the rouleaux content of the blood) and erythrocyte concentration (the more erythrocytes, the more rouleaux will be formed). I can now determine whether all rouleaux are interconnected and what kind of cohesive forces operate among them. It is known that at low yield velocities, a greater fraction of the erythrocytes form rouleaux [16]. These long columns of erythrocytes have a certain stiffness and might interweave to form a single structure [16]. It is hypothesized that cohesive forces may vary among rouleaux. This phenomenon makes the properties of blood resemble those of a solid body. When the yield velocity increases, the length of the rouleaux gradually decreases and ultimately only stand-alone erythrocytes are left.

To test this hypothesis, an experiment was conducted in which the breaking force and shear stress were those that naturally destroy rouleaux, but the cohesive forces were different. In an aerodynamic tube, a laminar boundary layer was created on a flat surface with the required shear stress on the surface of the wall [4]. On this surface, fine particles of equal diameter were placed (the cohesive force ranged from 0.0027 mN to 0.035 mN). From this information I could determine the destruction, i.e. the detachment and separation of particles from the surface. The results of the experiment are given in Table 3.

**Table 3:** The relationship between shear stress, particle diameter and damage to the wall

**Shear stress (N/m^2^)**	**Diameter of particles (mm)**	**Damage (g/s)**
0.043	0.25–0.63	0.002
0.051	0.25–0.63	0.03
0.092	0.25–0.63	0.07
0.13	0.25–0.63	0.122
0.13	0.5–0.63	0.05
0.158	0.5–0.63	0.1

Table 3 shows that destruction of rouleaux decreases with increasing particle diameter (which means increasing cohesive force). Conversely, the destruction of rouleaux increases with increasing shear stress. It can be supposed that an increase in shear stress destroys rouleaux that have a cohesive force lower than the breaking force. A further increase in shear stress will lead to the destruction of rouleaux with a greater cohesive force.

### Summary of results

#### The following have been determined

1. The dependence of the viscosity of a mixture on volume fraction during sedimentation of a group of particles (forming no aggregates), confirmed by published experimental data [7] for volume fractions of the second phase (*f*_2_) up to 0.6.

2. The dependence of viscosity of a mixture on the volume fraction of erythrocytes during sedimentation of rouleaux when the yield velocity is zero.

3. Increase in the velocity of a mixture with an increasing concentration of erythrocytes when yield velocity is zero.

4. An increased erythrocyte concentration results in an increase of viscosity of the mixture, and an increase in shear stress results in a decrease of viscosity of the mixture.

5. The quantity of rouleaux depends on shear stress (the higher the shear stress, the fewer rouleaux in the blood) and erythrocyte concentration (the more erythrocytes, the more rouleaux are formed).

6. With an increase in shear stress, those rouleaux are destroyed whose cohesive force is weaker than the breaking force. A further increase in shear stress will start to destroy rouleaux that have a greater cohesive force.

## Discussion

The role of the non-Newtonian viscosity of blood has remained a continuing challenge. Currently, the apparent viscosity at very low shear rates is considered as "effectively infinite immediately before the substance yields and begins to flow" [17]. Traditionally, Casson or Herschel-Bulkley models are used to measure both the yield stress of blood and shear thinning viscosity [18]. Human blood however does not comply with Casson's equation at a very low shear rate [13]. Other attempts to obtain finite viscosity values failed to take into account the hydrodynamic interactions between particles, or the complications related to aggregates [2]. Although an attempt to estimate blood viscosity at a very low shear rate has been made, no study has estimated the viscosity of blood when yield velocity equals zero.

The mathematical model created in this study used the most fundamental differential equations that have ever been derived to estimate blood viscosity. Depending on erythrocyte concentration, this model estimates the blood viscosity at zero yield stress. It takes into account the following factors: (1) Erythrocytes sediment as a group and not as single particles; (2) Erythrocytes interact with each other; (3) Erythrocytes sediment as a rouleaux; (4) Such rouleaux sediment within an erythrocyte-containing medium.

In general, abnormal values of blood viscosity can be observed in such pathologies as cancer [19,20], peripheral vascular disease [19,20] and acute myocardial infarction [19,20]. Blood hyperviscosity may impair the circulation and cause ischemia and local necrosis through decreased capillary perfusion [21]. Blood hyperviscosity due to abnormal red cell aggregation has been found in patients with diabetes, hyperlipidemia and cancer [22]. Estimation of blood viscosity is, however, particularly important in trauma patients. It is known that blunt trauma to vascular walls may lead to conditions for boundary layer separation [4]. Physically, this can be explained as follows [12]: flow retarded at the surface has low kinetic energy and cannot enter the high pressure zone, therefore it separates from the vessel wall and moves into the inner flow. It should be noted that under normal physiological conditions, the boundary layer does not separate [16]. Shear stress in the zone of boundary layer separation is equal to zero [4]. Therefore, in accordance with the above, trauma may create transient conditions for the formation of rouleaux or for the interlacing of existing rouleaux that have formed in the flowing blood [16], since there is no breaking force at zero shear and yield velocity. A certain number of rouleaux can then enter the arterial branching zone, where the shear velocity and shear stress on the internal wall are low [16], and these rouleaux might attach to the vessel wall, potentially causing atheromatosis. Such arterial branching zones could also be injured by blunt forces, which will also lead to boundary layer separation [4]. Therefore, rouleaux will be formed with low shear velocity and low shear stress on the internal wall [16], also creating conditions for atheromatosis.

Therefore, our understanding of the mechanism of blunt trauma to the vascular wall, which takes into account local hemodynamic and rheological factors, can be summarized in the following way. Trauma leads to the appearance of zones with high shear stress (as the result of injury to part of the vessel) and low or zero shear stress (within the zone of boundary layer separation) [4]. We have reported that high shear stress (exceeding the physiological value) may potentially damage the endothelium [4] and increase platelet aggregation [23,24], possibly leading to thrombus formation. On the other hand, trauma may lead to boundary layer separation, resulting in the appearance of a zone with zero shear stress and zero yield velocity [4]. This may result, according to current research, in an increase of blood viscosity through increased erythrocyte aggregation and rouleaux formation. Such hyperviscosity has been reported in patients with traumatic crush syndrome and also has been studied in animals exposed to traumatic crush [25]. As noted above, hyperviscosity may worsen the blood circulation and cause ischemia and local necrosis through deterioration in capillary perfusion [21].

This work also establishes a quantitative relationship between the extent of rouleaux formation and shear stress. According to current results, the number of rouleaux increases with decreasing shear stress, and this trend becomes more pronounced as the shear stress approaches zero. Rouleaux continue to form inside what I call the "hemodynamic shade". This "hemodynamic shade" creates a stagnant zone that can be characterized by a secondary flow and a boundary. Hemodynamic stress outside this zone, however, is still significant enough to destroy and entrain rouleaux. The "hemodynamic shade" zone can also be characterized by a significant deterioration of mass exchange due to the attachment of rouleaux to the vessel wall. This may decrease the permeability of the endothelium [16] and decrease the rate of removal of lipids and lipoproteins, which in turn can lead to the formation of lipid stripes directed along the blood flow and located in the "hemodynamic shade" of the original attached rouleaux. The escalating formation of rouleaux continues within the entire "hemodymanic shade" zone.

The model of traumatic damage to the vessel that takes into account local rheological and hemodynamic factors could be applied to many internal injuries involving an elastic vessel wall and a blunt traumatic mechanism. One example is traumatic myocardial infarction, which can result from blunt trauma to the coronary vessels. It should be noted that patients with blunt trauma may develop acute myocardial infarction; such patients may benefit from screening procedures such as electrocardiography, which might improve their chances of survival [8,26-49]. In a large cross-sectional observational study, abdominal, pelvic and blunt cardiac injuries were found to be significantly associated with acute myocardial infarction even after controlling for confounders such as mechanism and severity of injury, age, sex, race, source of payment, alcohol and cocaine use [50]. Intracoronary thrombosis has been suggested as one of the mechanisms of acute myocardial infarction in young people due to trauma, since other "atherosclerotic" mechanisms do not apply [38,42]. Nonetheless, the exact mechanism of traumatic myocardial infarction remains unclear. Current research suggests that blunt trauma may result in the appearance of a region of very low or zero shear stress, where hyperviscosity and increased rouleaux formation are likely to appear. Large quantities of rouleaux may be transported in the bloodstream toward the more distal parts of the coronary vessels, causing their occlusion. Caimi et al. [51], for instance, observed that blood viscosity at low shear rate is the only hemorheological factor that significantly increases the risk of acute myocardial infarction in young people. On the other hand, blunt trauma may result in traumatic compression of the vessel wall with high shear stress [4]. Increased shear stress itself may cause rupture of a coronary atherosclerotic plaque [52]. In addition, high shear stress may result in increased platelet aggregation [23,24], often leading to thrombus formation.

In summary, there is still a gap in our understanding of all quantitative aspects of the extreme values of viscosity at low and zero shear rates [3]. To the best of my knowledge, the work described in this paper represents one of the few attempts to estimate extreme values of viscosity at low shear rate. An understanding of the precise mechanisms that affect blood viscosity would be of clinical significance.
